# Efflux pump activity, biofilm formation and antibiotic resistance profile of *Klebsiella* spp. isolated from clinical samples at Lagos University Teaching Hospital

**DOI:** 10.1186/s13104-020-05105-2

**Published:** 2020-05-26

**Authors:** Sharon Akinpelu, Abraham Ajayi, Stella I. Smith, Adeyemi I. Adeleye

**Affiliations:** 1 Department of Microbiology, University of Lagos, Lagos, Nigeria; 2 Molecular Biology and Biotechnology Department, Nigerian Institute of Medical Research, Yaba, Lagos, Nigeria

**Keywords:** Efflux pump, Biofilm, Antibiotic resistance, *Klebsiella*

## Abstract

**Objective:**

Nosocomial and community acquired multidrug resistant *Klebsiella* infections are wide spread resulting in high morbidity and mortality due to limited number of antibiotics treatment options. This study investigated efflux pump activity, biofilm forming potential and antibiotic susceptibility profile of *Klebsiella* spp. isolated from clinical samples in a tertiary hospital in Lagos Nigeria. Eighteen clinical *Klebsiella* spp. isolated from urine, blood and sputum were subjected to antibiotic susceptibility testing using the disc diffusion method. Efflux pump activity was evaluated by the ethidium bromide cartwheel method and biofilm forming ability was determined by the tissue culture plate technique.

**Results:**

All 18 (100%) *Klebsiella* isolates were resistant to cefuroxime, cefixime, amoxicillin − clavulanate, ampicillin + cloxacillin, cefotaxime, and imipenem. Seventeen (94.4%) were resistant to ofloxacin while sixteen (88.9%) were resistance to nalidixic acid, Gentamicin and levofloxacin. All *Klebsiella* isolates possessed active efflux pump with the ability to form biofilm. However, their biofilm forming capabilities varied as 4 (22.2%) were strong, 3 (16.7%) were moderate and 11 (61.1%) were weak biofilm formers. Findings in this study reveal multiple factors at play in mediating the high level of antibiotic resistance observed in *Klebsiella* isolates. Hence a multifaceted approach is advocated in managing the infections caused by the pathogen.

## Introduction

Among pathogenic Gram-negative bacteria that belong to the family Enterobacteriaece, the genus *Klebsiella* consist of species known to cause community-acquired and nosocomial infections including urinary tract infections (UTIs), pneumonia, sepsis and bloodstream infection. With the emergence of antibiotic resistant and hyper-virulent *Klebsiella* strains, infections they cause result in high morbidity and mortality majorly in high risk individuals including immunocompromised, neonates and the elderly [1–4]. Several studies have reported multidrug resistant *Klebsiella* strains isolated from clinical cases. Rath and Padhy [5], reported a high prevalence of multidrug resistant *K*. oxytoca and *K*. pneumonia isolated from an Indian teaching hospital and adjoining community. Strains were resistant to fluoroquinolones, carbapenem, third and fourth generation cephalosporins. In a similar study, Fadeyi et al. [6] in Nigeria reported *Klebsiella* spp. of clinical origin that displayed high resistance to ceftazidime (100%), cefotaxime (94.0%), ciprofloxacin (70.0%) and trimethoprim-sulphamethoxazole (92.0%). However, no resistance to carbapenem was recorded. Biofilm formation has been linked to antimicrobial resistance and persistence observed in several pathogenic Gram negative bacteria including *Klebsiella* species [7]. Biofilm is an aggregation of sessile microorganisms enclosed in a matrix of extracellular polymeric substances. Their prevalence in medical implants, living and dead tissues make infection they cause difficult and expensive to treat [8]. Another factor that mediates antibiotic resistance in bacteria is the possession of active efflux pumps. Efflux pump systems expel a large array of chemical compounds including antibiotics from bacteria cells that possess them in an energy dependent manner [9]. It has been reported that efflux pump activity is connected to biofilm formation with a whole resultant effect of amplified antibiotic resistance [8] (Reza et al. 2019). Hence this study seeks to evaluate the efflux pump activity, biofilm formation and antibiotic resistance profiles of *Klebsiella* spp. isolated from clinical samples in a tertiary hospital in Lagos Nigeria.

## Main text

A total of eighteen *Klebsiella* isolates were obtained from Lagos University Teaching Hospital, Idi Araba Lagos Nigeria. Isolates were re-identified using standard biochemical tests according to Cheesebrough [10].

### Methodology

Antimicrobial susceptibility testing was carried out according to European Committee on Antimicrobial Susceptibility Testing [11], guidelines using the disc diffusion method. Eleven antibiotics including cefuroxime, gentamicin, cefixime, ofloxacin, amoxicillin − clavulanate, nalidixic acid, cefotaxime, ampicilin + cloxacillin, nitrofurantoin, imipenem and levofloxacin were used. Using a sterile cotton swab, one or two colonies of isolates were emulsified in normal saline and adjusted to 0.5 McFarland standard which was then applied onto the surface of Muller-Hinton agar. Antibiotic discs were then applied and incubated at 37 °C for 24 h. *E. coli* ATCC 25922 was used as for quality control.

Efflux pump activity of isolates was determined according to the method of Martins et al. [9] using the ethidium bromide (EtBr) cartwheel method. Cell suspension of isolates with approximately 10^6^ cells per mL were streaked on Muller-Hinton agar plates containing 0 mg/L, 0.5 mg/L, 1 mg/l, 1.5 mg/L and 2 mg/L concentrations of EtBr and incubated at 37 °C for 24 h. After which bacteria culture were viewed under a UV trans-illuminator (Cleaver Scientific Ltd) for fluorescence. Agarose gels that contained EtBr were dried and put in plastic bags which were then collected by the waste disposal unit for onward disposal.

Ability of isolates to form biofilm was determined by the tissue culture plate technique of Stephanovic et al. [12]. Single colonies of each *Klebsiella* isolate were inoculated into brain heart infusion (BHI) broth (Oxoid, Basingstoke, UK) supplemented with 2% sucrose and 200 µL of bacterial suspension was loaded into the individual wells of 96-well microtiter plate. Two hundred microlitre of sterile BHI broth was used as negative control. After 24 h incubation at 37 °C content of each well was discarded and wells were washed three times with sterile deionized water to remove non-adherent bacteria. Wells were air-dried for 45 min and 200 µL of 0.1% (v/v) crystal violet solution was added to each well and incubated for 45 min at room temperature then wells were again washed four times with sterile deionized water. Thirty-three percent glacial acetic acid was added to wells to solubilize incorporated dye. The optical density (OD) of stained adherent bacteria was measured at 650 nm using the Emax^®^ Plus Microplate Reader (Molecular Devices San Jose, CA). The assay was performed in triplicate.

## Results

**Fig. 1:**
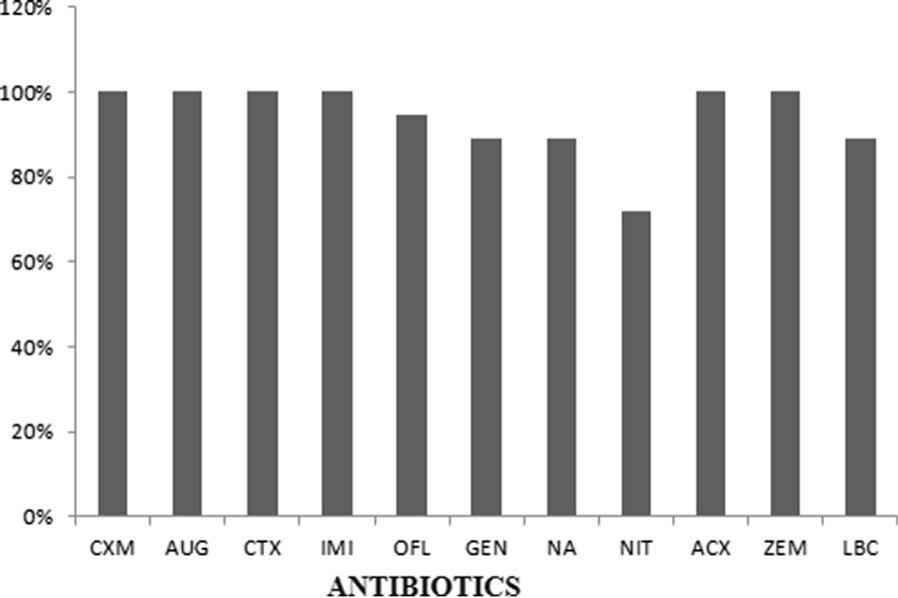
Percentage antibiotics resistance of *Klebsiella* clinical isolates. *CXM* cefuroxime, *GEN* gentamicin, *ZEM* cefixime, *OFL* ofloxacin, *AUG* amoxicillin − clavulanate, *NA* nalidixic acid, *CTX* cefotaxime, *ACX* ampicillin + cloxacillin, *NIT* nitrofurantoin, *IMI* imipenem, *LBC* levofloxacin

All eighteen (100%) *Klebsiella* isolates showed resistance to cefuroxime, cefixime, amoxicillin − clavulanate, ampicillin + cloxacillin, cefotaxime, and imipenem. Seventeen (94.4%) were resistant to ofloxacin while sixteen (88.9%) were resistance to nalidixic acid, gentamicin and levofloxacin. Only thirteen (72.2%) were resistant to nitrofurantoin as shown in Fig. 1.

All *Klebsiella* spp. isolates were positive for efflux pump activity as they fluorescence under UV light at 0.5 mg/L EtBr concentration. However only four (22.2%) were strong biofilm formers, while three (16.7%) and eleven (61.1%) were moderate and weak biofilm formers respectively. All strong biofilm formers were *Klebsiella* spp. isolated from urine, though others also isolated from urine were are moderate and weak biofilm formers as shown in Table 1.

**Table 1:** Biofilm forming potential, efflux pump activity and antibiotic susceptibility profile of *Klebsiella* spp

Isolate ID	Source	Biofilm forming potential			Efflux pump activity	Antibiotic resistance profile										
		Strong	Moderate	Weak	Phenotypic	CXM	AUG	CTX	IMI	OFL	GEN	NA	NIT	ACX	ZEM	LBC
K51	Urine	✓			+	R	R	R	R	R	R	R	R	R	R	R
K52	Urine			✓	+	R	R	R	R	R	R	R	R	R	R	R
K53	Urine			✓	+	R	R	R	R	R	R	R	R	R	R	R
K54	Urine			✓	+	R	R	R	R	R	S	R	R	R	R	R
K55	Urine		✓		+	R	R	R	R	R	R	R	S	R	R	R
K56	Urine		✓		+	R	R	R	R	R	R	R	R	R	R	R
K57	Urine			✓	+	R	R	R	R	R	S	R	R	R	R	R
K58	Urine			✓	+	R	R	R	R	R	R	R	S	R	R	R
K59	Urine			✓	+	R	R	R	R	R	R	R	R	R	R	R
K60	Urine	✓			+	R	R	R	R	S	R	S	R	R	R	S
K61	Urine			✓	+	R	R	R	R	R	R	R	S	R	R	S
K62	Blood		✓		+	R	R	R	R	R	R	R	S	R	R	R
K63	Sputum			✓	+	R	R	R	R	R	R	R	R	R	R	R
K64	Sputum			✓	+	R	R	R	R	R	R	R	R	R	R	R
K65	Urine			✓	+	R	R	R	R	R	R	R	R	R	S	R
K66	Urine			✓	+	R	R	R	R	R	R	R	R	R	R	R
K67	Urine	✓			+	R	R	R	R	R	R	R	S	R	R	R
K68	Urine	✓			+	R	R	R	R	R	R	R	R	R	R	R

*CXM* cefuroxime, *GEN* gentamicin, *ZEM* gefixime, *OFL* ofloxacin, *AUG* amoxicillin − clavulanate, *NA* nalidixic acid, *CTX* cefotaxime, *ACX* ampicillin + cloxacillin, *NIT* nitrofurantoin, *IMI* imipenem, *LBC* levofloxacin

Two isolates (K51 and K68) that were strong biofilm formers and exhibited efflux pump activity displayed pan resistance to all eleven test antibiotics. Although isolates K52, K53, K59 isolated from urine and isolates K63, K64 isolated from sputum that were weak biofilm formers with efflux pump activity were also resistant to all eleven test antibiotics.

## Discussion

Hospital and community acquired infections caused by *Klebsiella* spp. are wide spread and are becoming difficult to treat due to antibiotic resistance and biofilm formation ability of this pathogen [13, 14]. In this study *Klebsiella* spp. isolated from urine, blood and sputum showed high (100%) resistance to cefuroxime, cefixime, amoxicillin − clavulanate, ampicillin + cloxacillin, cefotaxime, and imipenem. Previous studies in Nigeria have also documented high and extensive resistance of *Klebsiella* spp. to β-lactam antibiotics [15–18]. Globally, resistance of *Klebsiella* spp. to β-lactam antibiotics including carbapenem is rife. Huai et al. [19] reported *Klebsiella pneumoniae* isolates that had high rate of resistance to impenem in China. Similarly, Yazdansetad et al. [20] a 50% rate of extended spectrum β-lactamase (ESBL) producing *K. pneumoniae* in Iran. In this study *Klebsiella* spp. also had considerable resistance to other antibiotics including ofloxacin (94.4%), nitrofurantoin (72.2%) and 88.9% resistance to nalidixic acid, gentamicin and levofloxacin. This is similar to the findings of Fadeyi et al. [6] and Humayun et al. [21] that reported the resistance of *Klebsiella* spp. to fluoroquinolone, aminoglycoside and sulfamethoxazole in Nigeria and Pakistan respectively. Several mechanisms have been deployed overtime by bacteria pathogens in evading the action of antibiotics. One of such mechanism is possessing active efflux pumps which was detected in all *Klebsiella* spp. isolates investigated in this study. Thus it could be asserted that active efflux pump played a role in the observed level of antibiotic resistance. Szabo et al. [22] and Maurya et al. [23] reported the role of efflux pump in quinolone and β-lactam antibiotic resistance in *Klebsiella pneumoniae* strains isolated from clinical samples in Hungary and India respectively. *Klebsiella* spp. are known to form biofilm which enhances their virulence and resistance to antibiotics [13]. Nirwati et al. [24] reported strong (26.95%), moderate (28.74%) and weak (29.94%) biofilm forming *K. pneumonia* isolates that were extensively resistant to antibiotics in tertiary care hospital in Indonesia. This is similar to our findings in this study although with slight variation of having 61.1% weak biofilm formers. However it was observed that the strength of biofilm formation be it strong, moderate or weak did not exclusively influence pan resistance to all test antibiotics used. This can be related to the study of Alcántar-Curiel et al. [25] in Mexico that reported no direct association of biofilm formation and ESBL production in multidrug resistant nosocomial *K. pneumonia* isolates.

## Conclusion

Multiple factors at play can generally amplify antibiotic resistance in bacterial pathogens as observed in this study. Hence there should be a multifaceted approach such as use of efflux pump inhibitors and biofilm disruptors in combination with antibiotic therapy in tackling the problem. Also routine surveillance should be a thing of priority in health care and community settings to curb the continuous transmission of antibiotic resistant *Klebsiella* spp.

## Limitation of study

It would be difficult to make far reaching conclusion as the number of isolates in this study are few. Hence future study should cover more hospital and investigate a larger number of isolates.

## Data Availability

All data obtained are available within the article.

## Abbreviations

ESBL: Extended spectrum beta lactamase
EtBr: Ethidium bromide
UV: Ultra violet
UTIs: Urinary tract infections
